# Responding to the COVID-19 pandemic in complex humanitarian crises

**DOI:** 10.1186/s12939-020-01162-y

**Published:** 2020-03-21

**Authors:** Danielle N. Poole, Daniel J. Escudero, Lawrence O. Gostin, David Leblang, Elizabeth A. Talbot

**Affiliations:** 1 Neukom Institute for Computational Science, Dartmouth College, Hanover, NH 03755 USA; 2 Department of Geography, Dartmouth College, Hanover, USA; 3 Department of Epidemiology, Dartmouth College, Hanover, USA; 4 Department of Epidemiology, Harvard T.H. Chan School of Public Health, Boston, USA; 5 Department of Mathematics, Dartmouth College, Hanover, USA; 6 Georgetown University Law, Washington, DC USA; 7 Department of Politics, University of Virginia, Charlottesville, USA; 8 Batten School of Leadership & Public Policy, University of Virginia, Charlottesville, USA; 9 Dartmouth Geisel School of Medicine, Hanover, USA

Over 168 million people across 50 countries are estimated to need humanitarian assistance in 2020 [1]. Response to epidemics in complex humanitarian crises—such as the recent cholera epidemic in Yemen and the Ebola epidemic in the Democratic Republic of Congo—is a global health challenge of increasing scale [2]. The thousands of Yemeni and Congolese who have died in these years-long epidemics demonstrate the difficulty of combatting even well-known pathogens in humanitarian settings. The novel severe acute respiratory syndrome coronavirus-2 (SARS-CoV-2) may represent a still greater threat to those in complex humanitarian crises, which lack the infrastructure, support, and health systems to mount a comprehensive response. Poor governance, public distrust, and political violence may further undermine interventions in these settings.

Populations affected by humanitarian crises are expected to be particularly susceptible to COVID-19, the disease caused by SARS-CoV-2, due to displacement, crowded housing, malnutrition, inadequate water, sanitation, and hygiene (WASH) tools, and stigmatization. Disease outbreaks further reduce access to limited healthcare, which is increasingly disrupted by attacks on health facilities and the persistent overburdening of health systems. These situations escalate both the necessity and the difficulty of delivering accurate and actionable information to potentially affected populations [3].

As the international community responds to SARS-CoV-2, public health authorities in humanitarian crises begin at a disadvantage to enact appropriate infection control to prevent transmission in healthcare settings, identify infectious cases, administer supportive care and novel treatments for the seriously ill, and trace contacts. These standard public health measures are particularly difficult to perform in humanitarian settings. For example, limited public health, laboratory, and primary care services represent a barrier to testing. Providing the limited healthcare worker cadre with appropriate training and personal protective equipment, and ensuring a continuous supply chain for such, is a challenge in all settings, exacerbated in complex humanitarian crises. Frequent displacement and limited contact information may prevent effective contact tracing. Finally, intractable structural challenges such as overcrowding limit the implementation of both quarantine of those exposed and isolation of those who are ill. Given these increased vulnerabilities, humanitarian crises should be viewed as a priority for national and international bodies that seek to combat this unfolding pandemic. Resources must be identified to protect healthcare workers, develop and deploy rapid testing, improve surveillance, and enact quarantine and isolation of contacts and cases.

To mitigate the impact of COVID-19 on crises-affected populations, governments and agencies will implement the familiar, global evidence-based approaches for combatting respiratory viruses. Respiratory hygiene is a highly effective public health intervention, supported by evidence demonstrating that the spread of respiratory viruses, such as SARS-CoV-2, can be prevented by hand hygiene, safe cough practice, and social distancing [4]**.** Hand hygiene is a readily implemented behavior: the distribution of soap to households in humanitarian settings has been shown to increase handwashing by over 30% [5]. Furthermore, hand hygiene is an avenue of agency for protecting one’s own health, consistent with the rights to dignity and to fully participate in decisions related to assistance in humanitarian crises. Widespread introduction of alcohol-based hand rubs is also possible in many resource-limited settings, with published protocols for local production [6].

The *Sphere Handbook*, a collection of rights-based guidelines for humanitarian response, is the foremost authority on minimum standards for humanitarian assistance [7]. However, despite the indisputable evidence for the efficacy of hand hygiene for reducing both bacterial and viral pathogen transmission, humanitarian WASH standards are based on evidence pertaining to the prevention of illnesses transmitted by the faecal-oral route, with the focus on hand hygiene proximate to latrines [5, 8]. And yet, latrines in crisis settings are often shared and distant from residential shelters, conferring a high risk of gender-based violence [9]. Gender-based violence around latrines is an important deterrent for accessing latrine-adjacent handwashing stations, particularly for hand hygiene to prevent respiratory pathogen transmission.

Evidence-based guidelines alone in complex humanitarian crises may not suffice during the emergence of the current SARS-CoV-2 pandemic. Without the adaptation of existing standards, mitigation plans will fall short of health and human rights obligations in outbreak response. Crisis-affected community engagement is integral in pandemic planning, in order to maximize the real-world effectiveness of efficacious interventions. Transparent and credible information-sharing mechanisms are increasingly essential when pandemics threaten vulnerable populations [10]. Diplomacy bridging long-standing mistrust of public health and biomedical interventions and facilitating engagement with contentious actors is a necessary component of effective health governance in complex crisis settings [2]. Interventions tailored to the needs of crisis-affected populations, delivered with transparent information, in the context of inclusive governance practices, are urgently needed in the global response to the COVID-19 pandemic.

## Data Availability

Not applicable.
